# Overview of innovative skill-mix changes in nursing across Europe

**DOI:** 10.1093/eurpub/ckac129.447

**Published:** 2022-10-25

**Authors:** C Maier, J Winkelmann, M Wismar

**Affiliations:** 1 Department of Healthcare Management, Technische Universität Berlin, Berlin, Germany; 2 European Observatory on Health Systems and Policies, Brussels, Belgium

## Abstract

**Background:**

Many countries in Europe and worldwide have implemented new professional for nurses or are in the process of implementation, yet, an overview of the evidence on skill-mix changes has been missing. This study has analysed skill-mix innovations in nursing, evidence on outcomes and lessons for implementation.

**Methods:**

An overview of systematic reviews, following a protocol plus country case studies, as part of an international study. The literature search was performed in six databases, with search terms covering skill-mix whereby the nursing profession played a key role. Screening was performed by three researchers after high interrater reliability rates were achieved. Analyses were performed for the nursing professions, a typology of skill-mix changes (task-shifting and role expansion) and evidence on outcomes.

**Results:**

A total of 42 systematic reviews were identified on nurses working in new roles. The roles varied considerably, ranging from nurse prescribing to advanced practice nursing and nurse-led clinics. Nurse-led chronic care programmes were frequently identified, with overall positive outcomes on several health outcomes, e.g., for patients with diabetes or cardiovascular diseases. Nurses were also working in advanced roles in health promotion and prevention, e.g., performing screening programmes, showing equivalent quality of care compared with doctors if adequately trained. Several skill-mix models to enhance care coordination and integration were identified, suggesting that when tailored to the needs of specific population groups, particularly for vulnerable groups, may improve health outcomes or access to services.

**Conclusions:**

The roles of nurses are increasingly diversifying and expanding internationally. Sharing country experiences on how to effectively educate the workforce to be prepared for these new roles and ensure smooth integration is critical.

